# Identification of Rotundone as an Important Contributor to the Flavor of Oak-Aged Spirits

**DOI:** 10.3390/molecules26144368

**Published:** 2021-07-20

**Authors:** Elizabeth Genthner-Kreger, Keith R. Cadwallader

**Affiliations:** 1 Sensient Flavors & Extracts, North America, 5115 Sedge Blvd, Hoffman Estates, IL 60192, USA; Elizabeth.Kreger@sensient.com; 2 Department of Food Science and Human Nutrition, University of Illinois, 1302 W. Pennsylvania Avenue, Urbana, IL 61801, USA

**Keywords:** flavor, rotundone, whiskey, gas chromatography–olfactometry, stable isotope dilution

## Abstract

Experiments were conducted to identify a compound responsible for a *spicy*, *woody*, *incense-like* odor note in oak-aged spirits. The target compound was extracted from oak wood and various oak-aged spirits and analyzed by multidimensional (heart-cut) gas chromatography–mass spectrometry–olfactometry (MD–GC–MS–O), and was unambiguously identified as the sesquiterpene ketone, 5-isopropenyl-3,8-dimethyl-3,4,5,6,7,8-hexadydro-1(*2H*)-azulenone (rotundone). Quantitation of the trace-level target compound was done by stable isotope dilution analysis (SIDA) in a variety of oak-aged spirits, including bourbon, rye, Tennessee whiskey, scotch, rum, and tequila. The content of rotundone was found to increase as a function of years of barrel aging for 4-, 8-, and 12-year-old bourbons obtained from the same manufacturer, thus confirming its origin to be from oak. In addition, odor-activity values (OAVs) were compared for selected potent odorants, including rotundone, in the same 4-, 8-, and 12-year-old bourbons, which indicated the relative importance of rotundone in the overall flavor of oak-aged spirits.

## 1. Introduction

The practice of barrel aging in the production of distilled beverages has been used for centuries. Oak wood is the wood of choice in barrel making for its physical characteristics that lend itself to manufacturing a barrel, and its unique chemical properties that impart desirable flavors, such *smoky*, *clove-*, and *vanilla-*like notes. Volatiles from oak wood are either naturally present in the wood or formed during the post-harvest treatment. Seasoning, the first step of post-treatment, is performed to dry the wood, which equilibrates the moisture content to prevent further shrinkage or swelling, and to prepare the wood for toasting/charring. Due to loss of moisture, seasoning also creates a higher concentration of volatile constituents in the wood. The subsequent toasting/charring step affects the volatile composition of oak through hydrothermolysis, during which lignin pyrolysis produces the more familiar flavors of oak aging including guaiacol, 4-ethylguaiacol, and 4-vinylguaiacol (*smoky*), eugenol and isoeugenol (*spicy, clove-like*), syringol and syringaldehyde (*sweet, smoky*), *p*-cresol (*barnyard, bandaid-like*), and vanillin (*vanilla*). Carotenoids, which are unique to oak wood, include both β-carotene and lutein, which break down to form volatile compounds such as β-ionone, β-damascenone, dihydroactinolide, and megastigmatrienones [1,2]. Lipids and carbohydrate go through reactions to produce volatile aldehydes, alcohols, esters, furans, lactones, and, most importantly, (*Z*)*-* and (*E*)*-*β-methyl-γ-octalactone, i.e., “oak lactones” or “whiskey lactones” [3,4].

The starting grain and subsequent treatment post-distillation, i.e., oak aging, are the two main contributors to the flavor of whiskey and other oak-aged spirits. With whiskey, the starting grain, typically barley, corn, wheat, or rye, goes through a malting step in which the grain partially germinates. Moreover, during the malting process, volatiles are formed via Maillard reactions, resulting in a product very similar to un-hopped beer [5]. The aroma impact compounds from fermentation consist of fusel alcohols, acetates, and esters, which impart fruity or solvent-like characteristics, and include 2-methyl-1-propanol, 2- and 3-methyl-1-butanol, 2- and 3-methylbutyl acetate, acetaldehyde, 2-methylpropyl acetate. Post-distillation oak aging is the most important step in developing the flavor of whiskey. The flavor compounds from the oak, as discussed previously, are directly extracted into the spirit as well as developed from the ethanolysis of the acids present. The latter process results in the formation of fruity esters, including ethyl propionate, ethyl butanoate, ethyl hexanoate, and ethyl octanoate along with several branched-chain ethyl esters.

Rotundone was at one time a fairly unassuming compound, first noted as a very potent aroma reminiscent of incense or black pepper, where it was first isolated from the root of *Cyperus rotundus* [6] and not mentioned again in the flavor science field until 2008, where it was identified as a potent odorant in Syrah grapes and wine [7]. It was again identified in agarwood oil, a dark fragrant resinous material from the heartwood of trees in the genus *Aquilaria*, a very prized and expensive material used in perfumery, described as warm, sandalwood, rich, woody, and ambergris [8]. The discovery of rotundone in grapes prompted its identification and quantitation in other food matrices [9]. Recently, it was reported as a character-impact odorant in chicory coffee [10]. Rotundone has an extremely low odor detection threshold of 8 parts-per-trillion (pptr) in water or 22 pptr in wine [7]. Therefore, even when present at low concentrations, rotundone may serve as an extremely potent odorant.

Despite extensive research on aroma active compounds, the identity of a component responsible for the “woodiness” in oak-aged spirits remains unknown [11,12]. Previous research cited the presence of an unknown compound with a *spicy, woody, incense-like* character, with the need to identify it [13,14,15]. The objectives of this study were to unambiguously identify and quantitate this unknown *spicy, woody, incense-like* compound and to determine its potential influence on the overall flavor in oak-aged spirits.

## 2. Results and Discussion

### 2.1. Identification of An Unknown “Spicy, Woody, Incense” Odorant in Oak Wood

A total of 30 odorants were detected by gas chromatography–olfactometry (GC–O) of the oak wood extract (Table 1). All identified compounds agreed with previous studies on volatiles of oak wood [16,17,18,19,20]. Amongst the compounds detected, one unknown (the target compound) odorant was described as *spicy, woody, incense-like*. To obtain an interpretable electron-impact mass spectrum (EI-M) of the target compound, the RI range was cut to both an odor detection port/flame ionization detector (ODP/FID) and to a mass selective detector (MSD). During the cut to the ODP, the target compound was marked, and then overlaid with the cut to the MSD. This was repeated using three configurations of different polarity columns. Only certain mass ions were consistently present on all marked spots of every configuration; in particular, *m/z* 218 was an indication of the molecular weight of the target compound. As a result of the characteristic odor description and mass of the target compound, an investigation of different wood oils, tobacco, hops, dried herbs, and roots was undertaken to determine if the *spicy, woody, incense-like* target compound was present in another source material. Extracts from Agarwood oil, white peppercorns, and the root from Cyperus rotundus contained an intense *spicy, woody, incense* peak at the same retention times as the target compound. Further analysis of Cyperus rotundus root enabled the identification of the target compound as 5-isopropenyl-3,8-dimethyl-3,4,5,6,7,8-hexa-hydro-1(2*H*)-azulenone (rotundone). The identification was confirmed by comparison to the authentic standard. Rotundone went largely went unnoticed as a potent flavor compound, except for being used in perfumery. Most recently, however, it was noted as potent odorant in grapes, as well as the reason behind the black pepper note in wine [7]. It was also identified in a number of products including black pepper, marjoram, geranium, rosemary, saltbush, basil, thyme, and oregano [21] and as chicory coffee [10]. Its potency was confirmed by determination of its odor detection threshold of only 8 ng/L in water [7]. The discovery of rotundone in oak wood prompted subsequent investigations in oak-aged products.

### 2.2. Quantitation of Rotundone

The results from rotundone quantitation in bourbons, and other aged spirits, are shown in Table 2. Results indicate that rotundone is, indeed, transferred from the oak wood into the distilled spirit, potentially having a significant impact on its flavor. Among the oak-aged samples analyzed, Johnnie Walker Black Label Scotch Whiskey and Appleton Estates Extra Rum had the lowest concentration at 0.150 µg/L and 0.152 µg/L, respectively, which is not surprising as both employ used whiskey barrels in the aging process. Scotch maturation is done in casks that are used, repaired, re-charred, and then reused [22]. Rum has no legal requirements as to the cask used in aging, but it is required that casks be coded according to their origin or previous history, i.e., “F1” and “F2” for fresh first and second fill, respectively, and “UR” for an unclassified refill [23].

Based on the quantitation results in the other samples, it was revealed that age is apparently not the only factor contributing to rotundone concentration considering that 12-year-old bourbon (Jim Beam) had a lower concentration (0.342 µg/L) than a 6-year-old (0.694 µg/L) and a 10-year-old Bourbon (1.35 µg/L) obtained from a different distillery (Bulleit). The rye from Bulleit contained one of the highest rotundone concentrations among the whiskeys evaluated, suggesting that factors in their manufacturing process, other than age, may have had an effect.

Climate humidity and temperature may influence volatile extraction during oak-aging of spirits. Generally, in lower-humidity climates, water evaporates from casks faster, resulting in spirits with higher ethanol content and a higher concentration of extracted volatiles [24]. Aging at higher temperatures was also reported to result in an increase in oak volatiles in the spirit [23]. In addition to environmental conditions, it is important to consider that three different varieties of American white oak may be used for cooperage, which will likely influence the concentration of rotundone in a particular sprit. This is supported by a study which showed that the geographical origin of the trees used for barrels, seasoning of the wood, and the coopering method all have an effect on the volatiles in wine [25]. Additionally, barrel size was shown to affect extraction rates, where the lower volume to wood surface area resulted in more concentrated volatiles from oak in wine [26,27].

Conclusive statements concerning the concentration of rotundone and aging time can be drawn from the data obtained in the current study, as demonstrated both in the Jim Beam and Bulleit bourbons. The Bulleit bourbons almost doubled the rotundone during aging from 0.694 µg/L (6 year) to 1.35 µg/L (10 year). The Jim Beam bourbons also showed an increase, although not as great, from 0.342 µg/L (4 year) to 0.403 µg/L (8 year) to 0.453 (12 year) (Table 2). 

One of the more interesting observations was that rotundone was present not only in aged tequila but also in unaged silver tequila. This was further confirmed by direct injection GC–O analysis of the unaged tequila, in which an odorant was detected with the same retention index and odor property as rotundone (data not shown). Quite possibly, rotundone also originates from the agave used in tequila manufacturing. Agave leaves are known to contain a wide variety of monoterpenes and sequiterpenes. A study profiling the terpene content from a variety of agave leaves using GC–MS reported 32 terpenes in Agave tequilana [28]. This group demonstrated that terpenes were also in the final distilled spirit, tequila, by identifying 29 different monoterpenes and sequiterpene using GC–MS [29].

In the past few years, rotudone has gone from an obscure sesquiterpene ketone to being identified in an array of herbs, spices, fruits, chicory and now in oak wood. We can speculate that it probably exists in many more natural materials. The more well-known it becomes, the more likely it is to be viewed as a more common odorant. With the extremely low odor detection threshold of 8 ng/L, rotundone is also likely to be potent in anything in which it is found.

### 2.3. Relative Potency of Rotundone in Bourbon Whiskey

#### 2.3.1. Identification of Potent Odorants in Bourbon Whiskeys of Increasing Age (4, 8, and 12 Years) by Aroma Extract Dilution Analysis (AEDA)

A total of 40 odorants were identified by AEDA in solvent extracts of three bourbon whiskeys varying in age (Table 3). Results show that the three bourbons analyzed had similar rankings, based on flavor dilution factors, for the majority of the compounds detected. The results are also in agreement with previous studies on the flavor analysis of whiskey [12,30,31,32,33,34,35,36]. Almost all of the potent odorants identified were derived from the oak barrel during aging. The main exceptions were the branched short-chained alcohols, 2/3-methyl-1-butanol and phenethyl alcohol, which are products of fermentation.

The most potent odorants were consistent across all samples based on the results of AEDA on two different GC columns, regardless of aging time. The most potent odorants were vanillin, (*Z*)-whiskey lactone, syringol, 2-phenethylethanol, 2-/3-methyl-1-butanol, guaiacol, (*E*)-isoeugenol, eugenol, and (*E*)-β-damascenone. These were followed by mainly ethyl esters. The order of potency of these can vary between whiskeys as ethyl esters are formed during the aging process when the ethanol reacts with the wood acids. As these samples are of different ages, the amount of ethyl esters formed would be expected to differ and, thus, should not be consistent among the whiskeys.

The current study was the first to identify the presence of rotundone in bourbon whiskey. It ranked among the moderately potent odorants, being detected at log_3_FD factors between 2 and 4, thus indicating that it has some impact on the overall flavor. The remaining compounds identified are well-known constituents of oak-aged spirits.

#### 2.3.2. Concentrations and OAVs of Selected Potent Odorants in Bourbon Whiskeys of Increasing Age (4, 8, and 12 Years)

A total of 25 odorants identified by AEDA in bourbon whiskeys were quantitated by stable isotope dilution analysis (SIDA) (Table 4). Some interesting observations can be made with respect to aging based on the analysis of these 4-, 8-, and 12-year-old bourbons. Statistical analysis revealed that all 25 compounds varied significantly among bourbons. Sixteen compounds showed definite linear increases in concentrations as a function of age. Previously, the effect of aging on the concentration of volatiles in whiskey showed that ethyl esters (ethyl octanoate, ethyl hexanoate, ethyl butyrate, ethyl vanillate, and ethyl 3-methylbutanoate) increased over time [33], which illustrated that ethanolysis was not selective as to the acid backbone structure with which it reacts. In the present study, only ethyl butanoate and ethyl vanillate showed a linear increase in concentration from 4 to 8 to 12 years of aging. One might expect that a plateau would eventually be met by 12 years as the starting material for ethanolysis becomes depleted. However, the concentrations could also increase as a result of evaporation of both water and ethanol during aging, thus decreasing the total volume and subsequently increasing the concentrations of some odorants. This could explain the increase in concentration of the fusel alcohols (2-methyl-1-propanol and 2-/3-methyl-1-butanol).

A linear increase with time was also observed for the important oak wood extractives vanillin, guaiacol, syringaldehyde, (*E*)-isoeugenol, (*Z*)-whiskey lactone, and (*E*)-whiskey lactone. These compounds are derived directly from the oak wood, so a valid assumption may be that the longer the spirit is in contact with the wood, the greater the amount would be extracted. The whiskey lactones, in particular, are considered to be among the most important components of the oak influence on whiskey, and correlate with a positive assessment of whiskey flavor [4]. In the present study, vanillin had a high coefficient of correlation (1.0) with respect to its increase in concentration with aging. This is in agreement with previous reports in which vanillin was observed to form not only during the charring step of cask manufacturing, but also during aging by a hydrolytic mechanism. Whiskey, being slightly acidic, causes acid-catalyzed hydrolysis of the lignin during aging, resulting in the formation of vanillin [37]. The relationship between eugenol and isoeugenol is also of interest. Both display a linear trend during aging; however, they are inverse to one another, as eugenol is isomerized to isoeugenol by migration of the double bond. This conversion was previously observed in model studies involving the artificial aging of apple cider using oak chips [38]. Meanwhile, our compound of interest, rotundone, also increased linearly with whiskey age. Although rotundone was measured at the lowest concentration of all the compounds quantitated, this is not a direct reflection of its potency owing to its very low odor detection threshold.

When characterizing a volatile compound, it is common to calculate its odor-activity value (OAV), which is the ratio of concentration of an odorant to its odor detection threshold. OAVs calculated using published odor detection thresholds [4,39,40,41,42,43,44] are shown in Table 5. Generally, the odor of a compound with an OAV above 1 is considered to be detectable in the product. Of the compounds quantitated, 19 out of the 25 had OAVs above 1. All of the ethyl esters fell into this category, with the exception of ethyl vanillate. Of the oak-derived odorants, (*E*)-isoeugenol, guaiacol, eugenol, (*Z*)-whiskey lactone, vanillin, γ-nonalactone, syringol, and rotundone qualify as potential flavor contributors. OAVs for (*E*)-whiskey lactone, 4-ethylphenol, syringol, *p-*cresol, and syringaldehyde were all below 1. 

This is the first study in which rotundone was identified and quantitated in spirits. Although rotundone was measured in the parts-per-trillion range, its extremely low threshold value yielded an OAV well above 1, which increased with the age of the bourbon. Thus, it can be concluded that rotundone was clearly detectable and impacted the flavor of bourbon whiskeys. Future studies are needed, possibly employing aroma models and omission studies, to further demonstrate the degree with which rotundone contributes to whiskey flavor. Lastly, it would be interesting to see how the concentration of rotundone affects the overall flavor attributes of whiskey, and whether it conclusively increases the woody flavor descriptor in aged spirits as a function of increased concentration.

## 3. Materials and Methods

### 3.1. Chemicals and Reference Standards

General reagent-grade chemicals and authentic flavor standards were purchased from Sigma-Aldrich Co. (St. Louis, MO, USA) unless otherwise indicated. β-Damascenone was obtained from Firmenich (Geneva, Switzerland). (*Z*)-2-Nonenal [45], 2-acetyl-1-pyroline [46], dihydromaltol [47], and rotundone [48] were synthesized according to the literature cited. 

The following labeled compounds listed in Table 6 were obtained from commercial sources: [^2^H_3_]-guaiacol, [^2^H_3_]-*p-*cresol, d_7_-2-methyl-1-propanol and [2H_11_]-3-methyl-1-butanol (CDN Isotopes, Pointe-Claire, Quebec, Canada), and [^2^H_8_]-ethyl acetate (Aldrich).

Compounds in Table 6 were synthesized according to published procedures, as follows: [^2^H_4_]-rotundone [10], [1,2-^13^C_2_]-2-phenylethanol [49]; 3-methylbutyl-[^2^H_3_]-acetate, 4-hydroxy-3-[^2^H_3_],5-dimethoxybenzene ([^2^H_3_]-syringol), 4-[^2^H_5_]-ethylphenol, [^2^H_5_]-ethyl vanillate, ^2^H_2_-γ-nonalactone, and [^2^H_2_]-(*Z*)- and [^2^H_2_]-(*E*)-whiskey lactone [32]; 4-hydroxy-3-[^2^H_3_],5-dimethoxybenzaldehyde ([^2^H_3_]-syringaldehyde), [^2^H_5_]-ethyl butanoate, [^2^H_5_]-ethyl hexanoate, [^2^H_5_]-ethyl octanoate and [^2^H_5_]-ethyl 3-methylbutanoate and [36]; 4-hydroxy-3-[^2^H_3_]-methoxybenzaldehyde ([^2^H_3_]-vanillin) [50]; [^2^H_4_]-(*E*)-β-damascenone [51]; [1,2-^13^C_2_]-2-phenylethylacetate, 2-[^2^H_3_]-methoxy-4-(2-propenyl)phenol (^2^H_3_-eugenol), and 2-[^2^H_3_]-methoxy-4-propenylphenol (^2^H_3_-isoeugenol) [52].

### 3.2. Materials

Toasted oak was obtained from Oak Chips Inc. (Waverly, OH, USA). *Cyrpus rotundus* “whole herb” (dried root) was from Chinese Herbs Direct (Torrance, CA, USA). Ground white peppercorn, *Piper nigrum* (Spice Islands Trading Co., San Francisco, CA, USA), was purchased locally (Champaign, IL, USA).

All spirits were purchased from a local liquor store (Binny’s Beverage Depot, Champaign, IL, USA). These included the following bourbons: Jim Beam Bourbon (4 year), Jim Beam Black Bourbon (8 year), Jim Beam Signature Craft Bourbon (12 year), Bulleit Bourbon (at least 6 year), Bulleit Bourbon 10 year (10 year), Elijah Craig Bourbon (12 year), W.L Weller Bourbon (12 year). Other aged spirits were Jack Daniels Tennessee Whiskey (at least 4 year), Johnnie Walker Black Label Scotch Whiskey (at least 12 year), Bulleit Rye Whiskey (at least 4 year), Appleton Estates Extra Rum (12 year), Don Julio Añejo Tequila (18 month), and Milagro Tequila (unaged). Values in parentheses indicate the declared barrel age of the spirits. 

### 3.3. Analysis of Oak Wood Extracts 

#### 3.3.1. For Identification of Odorants

Oak wood was isolated by simultaneous distillation–solvent extraction (SDE) as previously described [53]. Toasted American white oak chips (100 g) were added to a 1 L round bottom flask containing 500 mL of odor-free, distilled-deionized water. Dichloromethane (50 mL) was used as the extraction solvent. Extraction was conducted for 3 h (total reflux time). The solvent extract was dried over anhydrous sodium sulfate (10 g) and concentrated to 1 mL using a Vigreux column (45 °C) followed by further concentration using a gentle stream of ultra-high-purity (UHP) N_2_ gas. 

#### 3.3.2. For Identification of “*Spicy, Woody, Incense-Like*” Unknown Odorant in Oak Extracts

Volatiles in oak wood were isolated by SDE as described above with some modifications. Oak chips were finely ground using a Thomas Wiley Mini Mill (Thomas Scientific, Swedesboro, NJ, USA) before adding 500 g into a 5 L round bottom flask containing 2 L odor-free distilled-deionized water. Dichloromethane (200 mL) was used as the extraction solvent (total reflux time 6 h). Extract concentration was performed as described above. The resulting extract was washed with 1 M NaOH (3 × 50 mL) to remove acids and phenolics before loading onto a water-cooled glass column (45 cm × 1.5 cm, filled with silica 60 Å (pre-baked at 180 °C, with 5% *w/w* water added and equilibrated post bake)) in n-pentane to a height of 23 cm. Using N_2_ gas, pressure (1 psi) was applied to the flash column and the extract fractionated by increasing polarity using a succession of five 50 mL pentane:diethyl (*v/v*) ether mixtures (100:0, 90:10, 85:15, 80:20, 75:25, 50:50). Fractions possessing a *spicy, woody, incense-like* aroma detected by GC–O were pooled. The flash column procedure was repeated four times to obtain an extract equivalent to 2 kg of ground oak wood chips.

#### 3.3.3. Multidimensional GC–MS–O 

A custom-built multidimension GC equipped with a Deans switch, in-oven cryotrap, and switching valve to direct flow to either the MS or olfactory detector port (ODT) was used to selectively analyze for the target compound. The entire system consisted of a 6890 GC (Agilent Technologies, Inc., Santa Clara, CA, USA) equipped with a flame ionization detector (FID) and ODP (Gerstel Inc., Linthicum, MD, USA), a 5973N mass selective detector (MSD, Agilent Technologies, Inc.), a Deans switch (Agilent Technologies, Inc.), a JAS CyroTrap (Joint Analytical Systems, Newark, DE, USA), and an electrically actuated two-position valve (Valco Instruments Co. Inc., Houston, TX, USA).

In initial analyses, the volatiles were sent directly to the ODP in order to determine the retention times and retention indices (RI) for the heart-cuts of the target odorant on three different polarity columns (Stabilwax, RTX-5, or RTX-1701; 30 m × 0.25 mm i.d. × 0.25 µm film, Restek, Bellefonte, PA, USA). Heart-cuts from the first column to the second column, of different polarity to obtain orthogonal chromatographic resolution, were made between RI 2200–2300 (Stabilwax), 1700–1800 (RTX-5), and 1800–1900 (RTX-1701). After the cut sections were sent to the MS, the valve was switched for a second run to send the cut section to the ODP/FID, where the retention time for the target compound was noted. The total ion chromatograph (TIC) of the cut section from the MS and the cut FID outputs were overlaid; the mass spectral data were then evaluated for the target peak. This experiment was repeated using six different column configurations, where valve position A directed the cut section to the MS, and position B directed the cut section to the FID/ODP. Instrumental conditions used for GC–O and GC–MS are described in later sections.

### 3.4. GC–O Analysis of Distilled Spirits

#### 3.4.1. Direct Solvent Extraction

In a 50 mL test tube, spirit sample (10 mL) was diluted to approx. 10% ABV with deodorized–distilled water (e.g., 40 mL H_2_O for 40% ABV spirit sample). Sample was extracted three times with dichloromethane (CH_2_Cl_2_, 3 × 2 mL). For each extraction, the tube was sealed with a PTFE-lined cap, shaken vigorously for 5 min, and then centrifuged at approx. 1500× *g* for 10 min. The pooled solvent extract (bottom CH_2_Cl_2_ layers) was dried over 2 g of anhydrous sodium sulfate and concentrated to 0.5 mL under a gentle stream of UHP N_2_ gas. Extract was transferred to a 2 mL sample vial equipped with a PTFE-lined cap and stored at −20 °C until analysis.

#### 3.4.2. Aroma Extract Dilution Analysis (AEDA)

The three bourbon samples subjected to AEDA were extracted as described in Section 3.4.1. AEDA was performed using a 1:3 (*v/v*) dilution series to obtain 1:3 (Log_3_FD = 1), 1:9 (Log_3_FD = 2), 1:27 (Log_3_FD = 3), 1:81 (Log_3_FD = 4), 1:243 (Log_3_FD = 5), 1:729 (Log_3_FD = 6), 1:2187 (Log_3_FD = 7), 1:6561 (Log_3_FD = 8) dilution ratios. GC–O evaluations were performed by three experienced panelists. Results were based on consensus scores from 2 out of the 3 panelists.

The GC–O system used for AEDA consisted of a 6890 GC (Agilent Technologies, Inc.) equipped with an ODP (Gersel). Extracts (Section 3.4.1) were injected in the cold splitless mode using a Gerstel PTV inlet (−50 °C initial temperature, 0.1 min delay, 12 °C/s ramp to 250 °C; 1.10 min splitless valve delay time). Separations were performed on RTX-Wax or RTX-5 (15 m × 0.54 mm i.d. × 1 µm df; Restek) capillary columns. The initial oven temperature was 35 °C. After 5 min, the oven temperature was increased at 10 °C/min to the final temperature of 225 °C and held for 20 min. The flow rate of helium carrier gas was 5 mL/min. Column effluent was split between the FID (250 °C) and ODP (250 °C). For AEDA, 1:3 *v/v* serial dilutions were prepared for each extract (in CH_2_Cl_2_). Each dilution was evaluated by three experienced panelists and results based on consensus scores.

#### 3.4.3. Gas Chromatography–Olfactometry–Mass Spectrometry (GC–MS–O)

The GC–MS–O system consisted of a 6890 GC/5973N mass selective detector (MSD) (Agilent Technologies, Inc.). Extracts were injected in the cold splitless mode using a Gerstel PTV inlet (−50 °C initial temperature, 0.1 min delay, 12 °C/s ramp to 250 °C; 1.10 min splitless valve delay time). Separations were done on a Stabilwax-DA or Rxi-5sil-MS (30 m × 0.25 mm i.d. × 0.25 µm df; Restek) capillary columns. The initial oven temperature was 35 °C. After 5 min, the oven temperature was increased at 10 °C/min to the final temperature of 225 °C and held for 20 min. The flow rate of helium carrier gas was 1 mL/min. The mass spectra were recorded in full scan mode (35–300 a.m.u., scan rate 5.27 scans/s, interface temperature 250 °C, and ionization energy −70 eV.). For GC–O, column effluent was split between the MSD (250 °C) and ODP (250 °C). 

#### 3.4.4. Compound Identification

Positive (confirmed) identification of a compound was based on comparison of its chromatographic performance (retention indices on two columns of different polarities), EI-mass spectrum, and odor properties (when appropriate) to those of an authentic reference standard. Whenever one or more of the above criteria were not met, the compound was considered tentatively identified.

### 3.5. Quantitative Analysis of Selected Compounds by Stable Isotope Dilution Analysis (SIDA)

#### 3.5.1. Headspace Solid-Phase Microextraction GC–MS Method

Whiskey sample (1 mL) plus 4 mL of deodorized–distilled water was pipetted into a 22 mL SPME vial and sealed with a PTFE-lined septum cap. Labeled internal standards described in Table 6 were spiked using 10 μL syringes by piercing the cap and introducing the standard solution directly into the sample matrix, followed by gentle mixing. HS-SPME was conducted using a CombiPal autosampler (Leap Technologies, Inc., Carrboro, NC, USA). Sample vial was pre-incubated at 60 °C (250 rpm agitation) for 10 min prior to exposing a SPME fiber (2 cm, 50/30 µm, DVB/carboxen/polydimethylsiloxane fiber; Supelco) to the vial headspace for 30 min. The fiber was then desorbed by hot split injection (injector temperature 260 °C; split vent flow 10 mL/min) into the GC–MS system. 

The GC–MS system and conditions were the same as described in Section 3.4.4. For the MSD, the mass spectra (from 35–300 a.m.u) were acquired in the scan/SIM mode with scan range 35 to 300 a.m.u. and electron multiplier voltage +300 eV above autotune. SIM ions were monitored (dwell set at 50 for all ions) according to the SIM/analyte groups given in Table 6.

Calibration solutions were prepared by combining varying levels of unlabeled target analytes with the labeled internal standards at the following mass ratios (analyte:IS): 1:6. 1:4, 1:2, 1:1, 2:1, 4:1, 6:1. Each solution was analyzed by direct injection GC–MS and response factors (R_f_) determined by linear regression of a plot of peak area ratios versus mass ratios (Table 6). Peak area ratios were used to estimate concentrations of target analytes as follows:

(1) $$\mathrm{Conc}\;\left(\mu g/\mathrm{mL}\right)=\left[{\mathrm{Peak}\;\mathrm{area}}_{\mathrm{target}}/{\mathrm{Peak}\;\mathrm{Area}}_{\mathrm{IS}}\right]\times \;\mathrm{Mass}\;{\left(\mu g\right)}_{\mathrm{IS}}\;÷\;\mathrm{Sample}\;\left(\mathrm{mL}\right)$$

#### 3.5.2. Direct Solvent Extraction (DSE)–GC–MS Method

In a 60 mL conical test tube, whiskey sample (10 mL) was spiked with various levels of labeled internal standards described in Table 6 and then diluted to 10% ABV with deodorized–distilled water. Dichloromethane (CH_2_Cl_2_, 2.25 mL or 3.0 g) was added and the tube sealed using PTFE-lined cap. The tube was shaken vigorously for 5 min and then centrifuged at 1500× *g*. The solvent (bottom CH_2_Cl_2_ layer) was dried over 0.5 g of anhydrous sodium sulfate and transferred to a 2 mL sample vial equipped with a PTFE-lined cap. Extracts were stored at −20 °C until analysis.

The GC–MS system used for analysis consisted of a 7890A GC (Agilent Technologies, Inc.)/Pegasus IV time-of-flight (TOF) MS (LECO Corporation). Extracts (2 μL) were injected in the cold splitless mode (−50 °C initial temperature, held 0.1 min, then ramped at 8 °C/sec to 200 °C, held for 5 min, then ramped to 250 °C and held there for remainder of run; 1.10 min splitless valve delay time). Separations were done on a Stabilwax capillary column (30 m × 0.25 mm i.d. × 0.25 µm df; Restek) using helium as the carrier gas (1 mL/min). The initial oven temperature was 40 °C. After 5 min, the oven temperature was increased at 4 °C/min to the final temperature of 225 °C and held for 30 min. The flow rate of helium carrier gas was 1 mL/min. For GC–(TOF) the mass spectra (from 35 to 300 a.m.u.) acquisition rate was 50 spectra/sec. GC–MS interface temperature was 230 °C, source temperature was 200 °C, and ionization energy −70 eV. 

Peak areas for selected (quantitation) ions of labeled IS and target analytes were determined using Leco Chroma TOF software (version 3.34). Mass ion area ratios and R_f_ values were used to calculate concentrations of target analytes as described in Section 3.5.1.

#### 3.5.3. Direct Injection GC–MS Method

In a 2 mL vial, whiskey sample (0.100 mL) plus 1.0 mL of ether was spiked with 10 μL of a mixed internal standard solution (32.9 mg/mL d_7_-2-methyl-1-propanol, 34.8 mg/mL d_11_-3-methyl-1-butanol, and 0.860 mg/mL ^13^C_2_-2-phenylethanol in ethanol). The mixture was analyzed by direct injection GC–MS as described below.

The GC–MS system consisted of a 6890N GC/5973N MSD (Agilent Technologies, Inc.). Extract (2 μL) was injected in the hot split mode (260 °C; 15 mL/min purge flow). Separations were performed on a Rxi-5MS-sil column (30 m length × 0.25 mm i.d. × 0.25 µm film thickness, Restek) at a helium flow of 1 mL/min. Oven temperature was programmed from 40 °C (5 min initial hold) to 240 °C (30 min final time) at a ramp rate of 6 °C/min. MSD parameters were as follows: capillary direct interface temperature, 250 °C, ionization energy, 70 eV, mass range 35–500 amu; EM voltage, stune = 300 V, scan rate, 5 scans/s. The MSD was operated in the SIM/SCAN mode to enable greater sensitivity. The following ions were recorded in the SIM mode (dwell set at 50): 55, 57, 62, 74, and 81. The mass ion peak area ratios and R_f_ values were used to calculate concentrations of target analytes as described in Section 3.5.1.

### 3.6. Statistical Analysis

Data were analyzed by one-way Analysis of Variance (ANOVA) for each compound concentration using the Minitab 16 program (Minitab Inc., State College, PA, USA). For attributes with significant differences across products, Fisher’s LSD was used for means separation, with reporting differences at α ≤ 0.05.

## Figures and Tables

**Table 1:** Odor-active compounds extracted ^a^ from toasted American and French oak woods.

Compound	Odor Description	RI (Rtx-5) ^b^	Detected by G–CO	
			French	American
hexenal	*green, cut-grass*	811	+	+
heptanal	*citrus, orange*	904	+	+
unknown	*earthy, mushroom*	912		+
3-octanol	*mushroom*	982	+	+
*o-*cresol *^c^*	*creosote, inky*	1062		+
*p-*cresol *^c^*	*barnyard, bandaid*	1083		+
guaiacol	*smokey*	1085	+	+
4-hydroxy-2,5-dimethyl-3(2*H*)-furanone ^c^	*caramel, burnt sugar*	1096	+	+
dihydromaltol ^c^	*caramel*	1101		+
maltol ^c^	*caramel*	1110		+
3-hydroxy-4,5-dimethyl-2(*5H*)-furanone ^c^	*curry, maple*	1114		+
(*Z*)-2-nonenal	*hay/stale*	1149	+	+
(*E,Z*)-2,6-nonadienal	*melon, cucumber*	1156	+	+
(*E*)-2-nonenal	*hay/stale*	1161	+	+
(*E,E*)-2,4-nonadienal	*fatty, fried*	1217		+
(*E*)-oak lactone	*herbaceous, coconut*	1294	+	+
4-ethylguaiacol	*smokey, cloves*	1274	+	+
thymol	*woody, thyme*	1293		+
*p-*vinylguaiacol	*smokey, cloves*	1321	+	+
(*Z*)-oak lactone	*herbaceous, coconut*	1331	+	+
Syringol	*smokey*	1356	+	+
(*E*)-2-undecenal ^c^	*fresh, fatty, cilantro*	1366		+
eugenol	*spicy, cloves*	1362	+	+
*γ-*nonalactone	*fruity, peach*	1368		+
vanillin	*vanilla*	1392	+	+
*γ-*decalactone	*fruity, peach*	1463		+
(*E*)-isoeugenol	*spicy, cloves*	1463	+	+
(*Z*)-6-dodecen-γ-lactone	*creamy, dairy*	1665	+	+
*δ-*decalactone	*floral, peach, coconut*	1501		+
*unknown*	*spicy, woody, incense*	1724	+	+

^a^ Extracts prepared according to method 3.3.1. ^b^ GC retention index. ^c^ Tentatively identified compound.

**Table 2:** Concentration of rotundone in oak-aged distilled spirits.

Product	Concentration (µg/L, ppb) ^a^	% RSD ^b^
Jim Beam Bourbon (4 year) (JB 4)	0.342 ^c^	0.54 ^c^
Jim Beam Black Bourbon (8 year) (JB 8)	0.403 ^c^	1.4 ^c^
Jim Beam Signature Craft (12 year) (JB 12)	0.453 ^c^	1.9 ^c^
Bulleit Bourbon (~6 year)	0.694	0.12
Bulleit Bourbon 10 (10 year)	1.35	0.85
W.L Weller Bourbon (12 year)	0.393	1.8
Elijah Craig Bourbon (12 year)	0.694	10
Bulleit Rye Whiskey (~4 year)	0.434	0.58
Jack Daniels Tennessee Whiskey	0.166	0.10
Johnnie Walker Black Label Scotch Whiskey (~12 year)	0.150	2.2
Appleton Estates Extra Rum (12 year)	0.152	1.8
Don Julio Añejo Tequila (18 month)	0.307	0.85
Milagro Silver Tequila	0.100	1.1

^a^ Average concentration (n = 2). ^b^ Percent relative standard deviation (n = 2). ^c^ Means and %RSD from triplicate determinations (n = 3).

**Table 3:** Potent odorants determined by aroma extract dilution analysis of bourbon whiskeys aged for 4, 8, and 12 years.

Compound	Odor Description	RI ^a^		Log_3_ FD ^b^					
				JB 4		JB 8		JB 12	
		Wax	Rtx5	Wax	Rxt5	Wax	Rtx5	Wax	Rtx5
vanillin	*vanilla*	2529	1413	6	5	7	5	8	5
(*Z*)-whiskey lactone	*herbaceous, coconut*	1949	1329	5	5	8	6	8	6
syringol	*smokey*	2241	1355	7	4	8	5	7	5
2/3-methyl-1-butanol	*chocolate, malty*	1212	836	8	5	7	5	7	5
2-phenethyl alcohol	*rosey, wine-like*	1900	1120	6	4	6	5	7	4
guaiacol	*smokey*	1854	1087	7	4	7	5	7	4
(*E*)-isoeugenol	*spicy, cloves*	2335	1459	6	3	6	3	6	4
eugenol	*spicy, cloves*	2154	1361	5	4	5	4	6	3
(*E*)-β-damascenone	*floral, cooked apple*	1823	1391	4	4	5	4	4	4
ethyl hexanoate	*fruity, berry*	1245	1001	4	3	4	3	4	5
acetal	*fruity*	927	728	4	4	4	4	4	4
ethyl 2-methylbutanoate	*fruity, berry*	1070	857	3	3	4	3	4	4
ethyl butanoate	*fruity, bubble gum*	1046	808	3	2	3	3	4	4
(*Z*)-2-nonenal ^c^	*hay, stale*	1505	1149	2	2	3	2	4	3
syringaldehyde	*smokey, vanilla*	2884	1671	4	3	4	3	3	3
ethyl vanillate	*spicy*	2642	1580	3	1	3	2	3	1
rotundone	*spicy, woody, incense*	2262	1720	2	2	4	4	3	3
4-ethylphenol	*barnyard, bandaid*	2167	1180	1	1	1	3	3	1
γ-nonalactone	*fruity, peach*	2020	1370	3	2	3	3	3	2
2-phenethyl acetate	*floral, rosy*	1812	1258	3	1	3	1	3	1
ethyl octanoate	*fruity*	1428	1193	3	1	3	1	3	2
2-methyl-1-propanol	*chocolate, malty*	1099	634	1	ND	1	ND	3	ND ^d^
2/3-methylbutanoic acid ^c^	*sweaty*	1662		3	ND	3	ND	3	ND
3-methylbutyl acetate	*fruity, banana*	1147	875	<1 ^e^	1	<1	1	2	2
unknown	*fruity*	1198		1	<1	1	ND	2	ND
2-acetyl-1-pyrroline ^c^	*roasty, cereal*	1314	1052	2	1	1	1	2	2
acetic acid	*pungent, vinegar*	1444	832	1	ND	<1	ND	2	1
(*E*)-2-nonenal ^c^	*hay, stale*	1537	1164	1	<1	2	1	2	1
butyric acid	*cheesy*	1619	853	2	1	2	<1	2	2
(*E,E*)-2,4-nonadienal	*fatty, fried*	1700	1157	2	<1	1	1	2	2
unknown	*cereal/burnt*	1801	1050	2	1	4	<1	4	2
(*E*)-whiskey lactone	*coconut*	1882	1291	2	1	2	1	2	2
4-vinylguaiacol ^c^	*smokey, spicy*	2174	1320	2	ND	1	1	2	2
unknown	*soapy*	2456	1555	1	ND	1	<1	2	<1
*p*-cresol	*barnyard, bandaid*	2074	1109	1	1	<1	3	1	3
(*Z*)-6-dodeceno-γ-lactone ^c^	*creamy, diary*	2384	1666	1	<1	<1	ND	1	1
1-octen-3-one ^c^	*metallic, mushroom*	1306	982	1	ND	<1	1	<1	2
γ-decalactone ^c^	*fruity, peach*	2139	1465	ND	ND	ND	ND	<1	<1
β-ionone ^c^	*floral, violet*		1427	ND	ND	ND	<1	ND	<1
4-ethyl guaiacol ^c^	*smokey, spicy*	1994	1283	<1	ND	<1	ND	<1	2

^a^ GC retention index. ^b^ Log_3_ flavor dilution factors determined on polar (wax) and nonpolar (Rtx5) columns from aroma extracts prepared according to method 3.4.1. For JB4, JB8, and JB12, refer to Table 2. ^c^ Tentatively identified compound. ^d^ Not detected. ^e^ Compound detected only in concentrated extract.

**Table 4:** Concentrations for selected odorants in bourbon whiskeys aged for 4, 8, and 12 years.

Compound	Concentration (µg/L, ppb)			Sig.^a^
	Jim Beam Bourbon (4 Year)	Jim Beam Black Bourbon (8 Year)	Jim Beam Signature Craft (12 Year)	
3-methyl-1-butanol	962,000 (±4.0%)	1,090,000 (±4.5%)	1,420,000 (±4.9%)	0.97
2-methyl-1-butanol	379,000 (±4.3%)	462,000 (±4.1%)	727,000 (±4.5%)	0.96
2-methyl-1-propanol	224,000 (±4.4%)	321,000 (±8.5%)	419,000 (±8.6%)	1.0
2-phenethyl alcohol	33,800 (±0.40%)	41,700 (±1.4%)	39,100 (±1.4%)	*
syringaldehyde	7530 (±3.5%)	10,400 (±0.54%)	14,400 (±3.0%)	1.0
acetal	5390 (±3.7%)	5360 (±5.6%)	5170 (±0.49%)	*
ethyl octanoate	4790 (±5.2%)	3260 (±1.8%)	5830 (±3.0%)	*
(*Z*)-whiskey lactone	2850 (±2.1%)	4330 (±2.3%)	5470 (±1.8%)	1.0
3-methylbutyl acetate	1910 (±0.66%)	973 (±2.7%)	1980 (±1.9%)	*
ethyl hexanoate	1500 (±2.8%)	1420 (±1.5%)	3110 (±2.8%)	*
4-ethyl phenol	72.7 (±0.71%)	58.4 (±0.82%)	91.2 (±9.1%)	*
vanillin	619 (±2.3%)	950 (±2.9%)	1410 (±1.9%)	1.0
2-phenethyl acetate	614 (±1.3%)	123 (±1.7%)	211 (±0.74%)	*
ethyl butyrate	458 (±1.0%)	740 (±2.2%)	1260 (±2.6%)	0.99
guaiacol	39.1 (±7.6%)	56.7 (±4.8%)	63.5 (±4.3%)	0.97
(*E*)*-*whiskey lactone	316 (±0.48%)	457 (±0.81%)	586 (±2.3%)	1.0
(*E*)-isoeugenol	306 (±2.4%)	368 (±3.5%)	416 (±7.7%)	1.0
eugenol	207 (±3.9%)	197 (±2.0%)	131 (±5.3%)	−0.920
syringol	205 (±14%)	265 (±20%)	219 (±4.8%)	*
γ-nonalactone	145 (±13%)	190 (±3.7%)	175 (±14%)	*
ethyl vanillate	127 (±14%)	299 (±3.9%)	572 (±17%)	0.99
ethyl 3-methylbutanoate	85.0 (±1.0%)	183 (±3.1%)	313 (±11%)	0.99
*p*-cresol	18.0 (±4.0%)	26.9 (±26%)	23.6 (±7.8%)	*
(*E*)-β-damascenone	6.56 (±2.1%)	3.58 (±2.6%)	3.62 (±2.1%)	*
rotundone	0.342 (±0.54%)	0.403 (±1.4%)	0.453 (±1.9%)	1.0

^a^ For each row, values are coefficients of correlation (R), representing change in concentration of an odorant as a function of declared age; * indicates a significant difference (*p* < 0.05), but no correlation exists with respect to change in odorant concentration as a function of declared age.

**Table 5:** Odor-activity values for selected odorants in bourbon whiskeys aged for 4, 8, and 12 years.

Compound	ODT (µg/L, ppb) ^a^	Odor-Activity Value ^b^		
		Jim Beam Bourbon (4 Year)	Jim Bean Black Bourbon (8 Year)	Jim Beam Signature Craft (12 Year)
ethyl 3-methylbutanoate	1.6 [35]	53	114	195
ethyl butanoate	9.5 [35]	48	78	133
ethyl hexanoate	30 [35]	50	47	104
(*E*)-isoeugenol	6 [39] ^c^	51	61	69
(*Z*)-whiskey lactone	67 [4]	43	65	82
guaiacol	9.2 [35]	43	61	69
rotundone	0.008 [7] ^d^	43	50	57
vanillin	22 [35]	28	43	64
ethyl octanoate	147 [35]	33	22	40
(*E*)-β-damascenone	0.14 [35]	47	26	26
eugenol	7.1 [35]	29	28	18
2-phenylethanol	2600 [35]	13	16	15
γ-nonalactone	21 [35]	6.9	9.0	8.3
3-methylbutylacetate	245 [35]	7.8	4.0	8.1
acetal	719 [35]	7.5	7.5	7.2
2-methyl-1-propanol	101,000 [40]	2.2	3.2	4.1
2-methyl-1-butanol	212,000 [35]	1.8	2.2	4.1
3-methyl-1-butanol	561,000 [35]	1.7	1.9	2.5
2-phenylethyl acetate	108 [35]	5.7	1.1	2.0
(*E*)-whiskey lactone	790 [4]	0.40	0.58	0.74
4-ethylphenol	170 [35]	0.43	0.34	0.54
syringol	580 [41]	0.35	0.46	0.38
*p*-cresol	81.6 [42]	0.22	0.33	0.29
syringaldehyde	50,000 [43] ^c^	0.15	0.21	0.29
ethyl vanillate	900 [44] ^c^	0.14	0.33	0.64

^a^ Odor detection threshold (ODT) in 40% *v/v* ethanol/water unless otherwise indicated (reference given in brackets). ^b^ Odor-activity value (OAV) for an odorant was calculated by dividing concentration by corresponding ODT. ^c^ ODT determined in 10% *v/v* ethanol/water. ^d^ ODT determined in water.

**Table 6:** Calibration table: target analytes, labeled internal standards, IS spike masses, sample volumes, MS ions, and response factors used for SIDA of whiskey.

Method	Target	Labeled IS	Mass IS (µg)	Sample (mL)	Response Factor, R_f_ *(ion_target_ vs. ion_IS_)*
SPME (3.5.1)	acetal	[^2^H_8_]-ethyl acetate	243	1.0	0.521 *(103 vs. 96)*
	ethyl butanoate	[^2^H_5_]-ethyl butanoate	1.51	1.0	0.771 *(88 vs. 93)*
	ethyl hexanoate	[^2^H_5_]-ethyl hexanoate	2.50	1.0	0.831 *(88 vs. 93)*
	ethyl octanoate	[^2^H_5_]-ethyl octanoate	2.10	1.0	0.797 *(88 vs. 93)*
	ethyl 3-methylbutanoate	[^2^H_5_]-ethyl 3-methyl butanoate	0.797	1.0	0.834 *(88 vs. 93)*
	ethyl vanillate	[^2^H_5_]-ethyl vanillate	0.168	1.0	0.954 *(196 vs. 201)*
	3-methylbutyl acetate	3-methylbutyl [^2^H_3_]-acetate	3.89	1.0	0.834 *(87 vs. 90)*
	2-phenylethyl acetate	[1,2-^13^C_2_]-2-phenylethyl acetate	0.111	1.0	0.932 *(104 vs. 106)*
	2-phenylethanol	[^13^C_2_]-2-phenylethanol	41.4	1.0	1.00 *(122 vs. 124)*
	β-damascenone	[^2^H_4_]-β-damascenone	0.0352	1.0	0.468 (*190 vs. 194*)
DSE (*3.5.2*)	(*E*)-whiskey lactone	[^2^H_2_]-(*E*)-whiskey lactone	3.37	1.0	0.827 (*99 vs. 101*)
	(*Z*)-whiskey lactone	[^2^H_2_]-(*Z*)-whiskey lactone	3.57	1.0	0.546 (*99 vs. 101*)
	γ-nonalactone	[^2^H_2_]-γ-nonalactone	0.204	1.0	1.11 (*85 vs. 87*)
	guaiacol	[^2^H_3_]-guaiacol	5.35	10.0	1.02 (*124 vs. 127*)
	*p-*cresol	[^2^H_8_]-*p-*cresol	0.306	10.0	0.552 (*107 vs. 113*)
	4-ethylphenol	[^2^H_5_]-4-ethylphenol	2.30	10.0	0.845 (*122 vs. 127*)
	syringol	[^2^H_3_]-syringol	13.3	10.0	0.933 (*154 vs. 157*)
	eugenol	[^2^H_3_]-eugenol	2.40	10.0	0.611 (*164 vs. 167*)
	(*E*)-isoeugenol	[^2^H_3_]-(*E*)-isoeugenol	2.44	10.0	0.932 (*164 vs. 167*)
	vanillin	[^2^H_3_]-vanillin	24.6	10.0	0.974 (*152 vs. 155*)
	syringaldehyde	[^2^H_3_]-syringaldehyde	175	10.0	0.611 (*182 vs. 185*)
	rotundone	[^2^H_4_]-rotundone	0.0104	10.0	0.998 (*218 vs. 206*)
Direct injection (*3.5.3*)	2-methyl-1-propanol	[^2^H_7_]-2-methyl-1-propanol	15.0	0.10	0.956 *(74 vs. 81)*
	2-methyl-1-butanol	[^2^H_11_]-3-methyl-1-butanol	15.8	0.10	0.963 *(57 vs. 62)*
	3-methyl-1-butanol				0.833 *(55 vs. 62)*

## Data Availability

Not applicable.

## Sample Availability

Samples of the compounds are not available from the authors.
